# Antiviral Activity of the Polyene Macrolide Roseofungin Against Influenza a Virus: In Vitro, In Ovo, and Preliminary In Vivo Evaluation

**DOI:** 10.3390/v18090941

**Published:** 2026-08-28

**Authors:** Andrey Bogoyavlenskiy, Vladimir Berezin, Pavel Alexyuk, Madina Alexyuk, Irina Zaitseva, Aidar Mukhametkaliyev, Yergali Moldakhanov, Elmira Anarkulova, Timur Kerimov, Vyacheslav Dushenkov

**Affiliations:** 1 Research and Production Center for Microbiology and Virology, Almaty 050010, Kazakhstan; vberezin359@gmail.com (V.B.); elya-111@mail.ru (E.A.);; 2 Faculty Veterinary and Zooengineering, Kazakh National Agrarian Research University, Almaty 050010, Kazakhstan; mukhametkalievaidar@gmail.com; 3 Department of Natural Sciences, Hostos Community College, City University of New York, Bronx, NY 10451, USA; vdushenkov@hostos.cuny.edu

**Keywords:** antiviral activity, roseofungin, polyene macrolide, Influenza A virus, neuraminidase, viral adsorption, oseltamivir resistance

## Abstract

Roseofungin, a polyene macrolide antibiotic produced by *Streptomyces roseoflavus*, was evaluated for antiviral activity against Influenza A virus using complementary in vitro, in ovo, and preliminary in vivo toxicity models, supported by molecular docking and molecular dynamics simulations. The compound exhibited antiviral activity against multiple influenza A virus subtypes, including an oseltamivir-resistant H1N1 strain, with EC_50_ values ranging from 2.0 to 2.5 μg/mL and selectivity indices of 20–25. Functional assays demonstrated that roseofungin interfered with early stages of the viral life cycle by reducing viral adsorption and partially inhibiting neuraminidase activity. In virucidal assays, treatment with roseofungin resulted in a 1.5–2.5 log_10_ reduction in infectious viral titers. Acute toxicity studies in mice indicated low toxicity under the experimental conditions (LD_50_ > 25 mg/kg). Molecular docking predicted favorable interactions of roseofungin with multiple influenza A virus proteins. Subsequent molecular dynamics simulations of the hemagglutinin–roseofungin complex maintained the stability of the predicted binding mode across three independent 50 ns simulations (150 ns cumulative). Collectively, these findings demonstrate that roseofungin possesses broad-spectrum antiviral activity against genetically diverse Influenza A virus strains and represents a promising lead compound for the development of novel membrane-active antiviral agents. Further studies are warranted to elucidate its molecular mechanism of action and to evaluate its therapeutic potential in vivo.

## 1. Introduction

Influenza A virus (IAV) remains one of the most important respiratory pathogens affecting both human and animal populations worldwide [1,2]. Seasonal influenza epidemics are responsible for substantial morbidity and mortality each year, while the continued emergence of novel influenza strains poses an ongoing threat of future pandemics [3]. Although vaccines and antiviral drugs have significantly reduced the burden of influenza, their effectiveness is compromised by continuous viral evolution, antigenic drift, and the emergence of drug-resistant variants [4,5]. Consequently, there is growing interest in identifying antiviral agents that act through mechanisms distinct from those of currently available neuraminidase inhibitors and polymerase-targeting drugs [6].

Multiple stages of the influenza A virus life cycle are governed by dynamic interactions with host cell membranes. Viral infection begins with the attachment of the viral hemagglutinin (HA) to sialic acid-containing receptors on the host cell surface, followed by receptor-mediated endocytosis and membrane fusion. Subsequent stages of the viral life cycle, including intracellular trafficking, virion assembly, and budding, also depend on coordinated interactions between viral proteins and host cellular membranes. Because the viral envelope is derived from the host plasma membrane, its lipid composition and physicochemical properties can substantially influence viral infectivity and replication efficiency [7,8]. Increasing evidence indicates that perturbation of membrane organization, particularly cholesterol-rich membrane microdomains, may interfere with multiple stages of the influenza virus life cycle without directly targeting rapidly evolving viral proteins [9].

The dependence of enveloped viruses on membrane-associated processes has stimulated interest in host-directed antiviral strategies that target viral envelopes, membrane organization, or host factors involved in virus–cell interactions. In contrast to conventional direct-acting antivirals, membrane-targeting compounds may inhibit conserved stages of viral infection shared among genetically diverse viral strains, potentially reducing the likelihood of resistance development. However, achieving sufficient antiviral selectivity while minimizing toxicity toward host cells remains a major challenge for this therapeutic approach.

**Figure 1:**
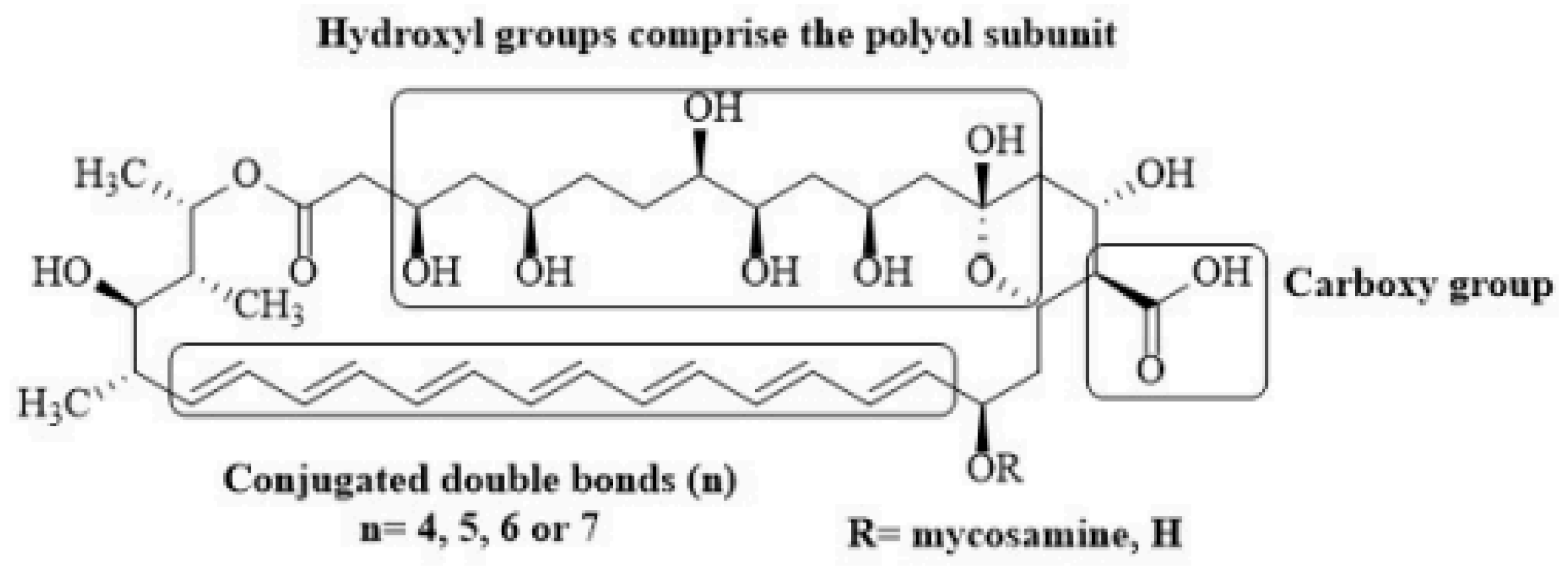
General structural features of polyene antibiotics [11].

Polyene macrolides are membrane-active natural products produced predominantly by *Streptomyces* species (Figure 1) [10,11].

These amphiphilic molecules contain a large macrolactone ring with multiple conjugated double bonds and hydroxyl groups that confer high affinity for membrane sterols [11]. Their selective interaction with membrane sterols, particularly ergosterol, underlies their well-established antifungal activity, as exemplified by amphotericin B and nystatin. Beyond antifungal applications, increasing evidence suggests that sterol-binding polyenes and other membrane-active natural products may also exhibit antiviral activity against several enveloped viruses by disrupting membrane-dependent stages of viral infection [12,13]. Nevertheless, the antiviral potential of this class of compounds remains insufficiently explored, and their mechanisms of action appear to vary among different viral systems.

Roseofungin is a naturally occurring polyene macrolide antibiotic produced by *Streptomyces roseoflavus* [14]. Structurally, it belongs to the carbonyl-conjugated pentaene subgroup of polyene macrolides and possesses amphipathic properties characteristic of membrane-interacting compounds. Previous studies have primarily focused on its antifungal activity, which has been attributed to sterol-dependent alterations in membrane integrity and permeability. In contrast, virtually no information is available regarding its antiviral activity against influenza viruses [15].

Although preliminary observations have suggested that roseofungin may inhibit influenza virus replication [16], its antiviral spectrum, efficacy against genetically diverse influenza A virus strains, selectivity, and potential mechanisms of action have not been systematically investigated. In particular, it remains unclear whether its antiviral activity results primarily from direct interactions with viral particles, interference with virus attachment and entry, modulation of membrane-associated processes, or a combination of these mechanisms.

In the present study, we evaluated the antiviral activity of roseofungin against a panel of Influenza A virus strains representing human, swine, and avian lineages using complementary in vitro and in ovo experimental models. We assessed its antiviral efficacy, cytotoxicity, selectivity, and effects on viral adsorption, neuraminidase activity, and viral infectivity. By establishing the antiviral profile of roseofungin across genetically diverse influenza viruses, this study provides a foundation for future investigations into its molecular mechanism of action and its potential development as a membrane-active antiviral agent.

## 2. Materials and Methods

### 2.1. Study Design

The study was designed to evaluate the antiviral activity, cytotoxicity, and preliminary safety profile of roseofungin using complementary in vitro, in ovo, and in vivo experimental systems. Antiviral efficacy was assessed in cell culture models of Influenza A virus infection and in embryonated chicken eggs, while cytotoxicity and acute toxicity were evaluated in mammalian cell lines, embryonated eggs, and mice.

Experimental groups included untreated controls, vehicle controls receiving phosphate-buffered saline (PBS) and treatment groups exposed to serial concentrations of roseofungin. Unless otherwise specified, all experiments were performed in at least three independent biological replicates.

The experimental unit was defined as a single well for cell culture assays, an individual embryonated egg for in ovo experiments, and an individual animal for in vivo studies.

### 2.2. Cell Lines and Culture Conditions

Madin–Darby canine kidney (MDCK), mouse liver (NCTC 1469), and sheep kidney (PO) cell lines were obtained from recognized biological repositories and maintained according to standard cell culture procedures.

MDCK cells were used for antiviral assays because of their established susceptibility to Influenza A virus infection. NCTC 1469 and PO cells were used for cytotoxicity assessment.

Cells were cultured at 37 °C in a humidified atmosphere containing 5% CO_2_ using the recommended growth media supplemented with 10% fetal bovine serum (FBS), 100 U/mL penicillin, and 100 μg/mL streptomycin. Cells were routinely passaged at 80–90% confluence and examined microscopically to confirm normal morphology before use in experiments.

### 2.3. Animals, Housing, and Ethical Approval

All animal procedures were conducted in accordance with institutional and national guidelines for the care and use of laboratory animals, and were approved by the Institutional Animal Care and Use Committee (Protocol No. 02-09-184; approved 30 October 2023).

Mice were housed in ventilated cages under controlled environmental conditions (22 ± 2 °C, relative humidity 50–60%, 12 h light/dark cycle) with unrestricted access to food and water throughout the study.

Embryonated chicken eggs (10 days of incubation) were maintained at 37 °C under controlled humidity conditions and candled daily to assess viability.

### 2.4. Sample Size Considerations

Sample sizes were selected based on previous studies employing comparable antiviral and toxicological experimental models and were considered sufficient for the exploratory evaluation of biological activity while minimizing animal use.

Cell culture experiments were performed using triplicate wells in at least three independent experiments. In ovo experiments utilized six embryonated eggs per treatment group, and acute toxicity studies included six animals per experimental group.

Because the study was exploratory in nature, no formal a priori statistical power calculation was performed.

### 2.5. Randomization

For in ovo and in vivo experiments, embryonated eggs and animals were randomly assigned to treatment groups using a computer-generated randomization procedure. Allocation was completed before treatment administration to minimize selection bias.

### 2.6. Blinding

Outcome assessment was conducted under partial blinding conditions whenever feasible.

Investigators performing MTT assays, RT-qPCR analyses, and hemagglutination assays were blinded to treatment allocation during data acquisition and analysis. Complete blinding was not possible during preparation and administration of compound dilutions.

Data processing and statistical analyses were performed using coded datasets to minimize observer bias.

### 2.7. Inclusion and Exclusion Criteria

The following predefined criteria were applied throughout the study:

- Cell culture assays: wells exhibiting contamination, abnormal morphology, or technical failure before treatment initiation were excluded.
- In ovo experiments: embryonated eggs that died within 24 h after inoculation were excluded as non-specific procedural losses.
- In vivo studies: animals displaying preexisting signs of illness or abnormal behavior prior to treatment were excluded.

All exclusions were documented prospectively. No outliers were removed after the completion of experiments.

### 2.8. Virus Strains and Propagation

The following Influenza A virus strains were used: A/Vladivostok/2/09 (H1N1, oseltamivir-resistant), A/Almaty/8/1998 (H3N2), A/Almaty/04/19 (H1N1), A/Swine/Iowa/1/30 (H1N1), A/Tern/South Africa/1/61 (H5N3), A/Chicken/FPV/Rostock/1934 (H7N1), and A/Chicken/Almaty/220/20 (H9N2).

Virus stocks were obtained from established virus repositories and propagated either in MDCK cell cultures or by inoculation into the allantoic cavity of 10-day-old embryonated chicken eggs depending on strain characteristics.

Viral infectivity titers were determined by observation of cytopathic effect (CPE), hemagglutination assay, or endpoint titration and were expressed as TCID_50_/mL or EID_50_/mL [17]. Median infectious doses were calculated using the Reed–Muench method [18].

### 2.9. Macrolide Antibiotic

Roseofungin used in the present study was isolated from *Streptomyces roseoflavus* var. *roseofungini* AS-20.14 and was characterized by physicochemical and spectroscopic methods, as described in our recent study [19]. The characterization included UV–Vis spectrophotometry, IR spectroscopy, NMR spectroscopy, thermogravimetric analysis (TGA), and differential scanning calorimetry (DSC), supporting the identity and physicochemical characteristics of the compound. Roseofungin was obtained from the Research and Production Center for Microbiology and Virology (Almaty, Kazakhstan). Roseofungin (CAS Number: 12687-98-8; molecular formula: C_39_H_62_O_10_; molecular weight: 690.9 g/mol; class: polyenic macrolide antibiotic, carbonyl-conjugated pentaene subgroup), HY-123424 from MedChemExpress (Monmouth Junction, NJ, USA) and T26124 from TargetMol (Wellesley Hills, MA, USA) [20].

### 2.10. Cytotoxicity Assay

Cell viability was evaluated using the MTT colorimetric assay. Cells were seeded into 96-well tissue culture plates at a density of 1 × 10^4^ cells per well and allowed to adhere for 24 h.

Roseofungin was prepared as serial two-fold dilutions ranging from 1 to 128 μg/mL. Following removal of the growth medium, cells were exposed to the respective concentrations of roseofungin for 48 h at 37 °C in a humidified atmosphere containing 5% CO_2_.

After incubation, the culture medium was replaced with fresh medium containing MTT (0.5 mg/mL), and cells were incubated for an additional 3 h. Formazan crystals were dissolved in dimethyl sulfoxide (DMSO), and absorbance was measured at 540 nm using a microplate reader.

Cell viability was expressed as a percentage of untreated controls. The 50% cytotoxic concentration (CC_50_) was calculated by nonlinear regression analysis of concentration–response curves [21].

### 2.11. Embryotoxicity Assay

Embryotoxicity was evaluated in 10-day-old embryonated chicken eggs.

Serial dilutions of roseofungin were inoculated into the allantoic cavity (100 μL per egg; *n* = 6 per group). Egg viability was monitored twice daily for five consecutive days by candling.

The median embryolethal dose (ELD_50_) was estimated using the Reed–Muench method [18].

### 2.12. Acute Toxicity Study

Acute toxicity was assessed in mice following a single intragastric administration of roseofungin.

Animals received 100 μL of the designated test solution and were monitored daily for 14 days. Clinical observations included changes in behavior, activity, appearance, body weight, and survival.

Humane endpoints were established before study initiation, and animals exhibiting severe distress were euthanized according to institutional animal welfare guidelines.

The median lethal dose (LD_50_) was estimated using the Reed–Muench method when applicable [18].

### 2.13. Viral Adsorption Inhibition Assay

To evaluate the effect of roseofungin on early virus–cell interactions, influenza virus suspensions were pre-incubated with serial concentrations of roseofungin for 30 min at room temperature before addition to confluent MDCK cell monolayers.

Following the adsorption period, cell monolayers were washed thoroughly with phosphate-buffered saline to remove unbound virus particles. Total cellular RNA was subsequently extracted and analyzed by quantitative reverse-transcription PCR (RT-qPCR).

The inhibitory concentration required to reduce cell-associated viral RNA by 50% (IC_50_) was calculated from concentration–response curves.

### 2.14. RNA Extraction and Quantitative PCR

Total RNA was isolated using a silica membrane-based purification system according to the manufacturer’s instructions.

Reverse transcription and quantitative PCR were performed using TaqMan chemistry on a QuantStudio 5 Real-Time PCR System. Amplification targeted the highly conserved matrix (M) gene of Influenza A virus [22].

The following primers and probes were used:

Forward primer: 5′-AGA TGA GTC TTC TAA CCG AGG TCG-3′

Reverse primer: 5′-TGC AAA AAC ATC TTC AAG TCT CTG-3′

Probe: 5′-[FAM]-TCA GGC CCC CTC AAA GCC GA-[TAMRA]-3′ [22].

Relative viral RNA levels were calculated using the comparative Ct (2^−^ΔΔCt) method and normalized to untreated infected controls [23].

### 2.15. Neuraminidase Inhibition Assay

Neuraminidase activity was evaluated using a fluorometric assay based on the substrate 2′-(4-methylumbelliferyl)-α-D-N-acetylneuraminic acid (MUNANA).

Virus suspensions were incubated with serial concentrations of roseofungin before the addition of substrate. Fluorescence intensity was measured according to the manufacturer’s protocol.

Results were expressed as percentage inhibition relative to untreated virus controls. The concentration producing 50% inhibition of neuraminidase activity (IC_50_) was determined by nonlinear regression analysis.

### 2.16. Antiviral Activity Assay

Antiviral activity was assessed using virus-induced cytopathic effect (CPE) inhibition in MDCK cell cultures.

Influenza virus suspensions were incubated with serial dilutions of roseofungin for 30 min before inoculation onto cell monolayers. Following adsorption, cultures were maintained in infection medium containing the corresponding concentration of roseofungin throughout the experimental period.

After incubation, antiviral efficacy was quantified using the MTT assay.

The effective concentration required to reduce virus-induced cytopathic effects by 50% (EC_50_) was calculated by nonlinear regression analysis. The selectivity index (SI) was determined as the ratio of CC_50_ to EC_50_ [21].

### 2.17. In Ovo Antiviral Assay

For evaluation of antiviral activity in embryonated eggs, virus–compound mixtures were incubated for 30 min before inoculation into the allantoic cavity of 10-day-old embryonated chicken eggs.

Following incubation, allantoic fluids were harvested, and viral replication was assessed by hemagglutination assay.

Antiviral activity was expressed as the reduction in hemagglutination titers relative to untreated infected controls [24,25,26].

### 2.18. Virucidal Assay

Direct effects of roseofungin on viral infectivity were assessed by incubating virus suspensions with roseofungin for 30 min at 37 °C.

Following incubation, samples were diluted before titration to minimize potential carry-over effects of residual compound during infectivity determination.

In the present study, roseofungin was dissolved in 50% (*v*/*v*) ethanol to prepare the stock solution. Serial working dilutions were subsequently prepared in the appropriate assay medium. The corresponding ethanol-containing vehicle control was included in the experiments at the same final solvent concentration as that present in the roseofungin-treated samples.

Briefly, 100 µL of roseofungin at 32 µg/mL was mixed with 100 µL of virus suspension, resulting in a two-fold reduction in the roseofungin concentration to 16 µg/mL in the treatment mixture. Following the treatment, the samples were subjected to serial 10-fold dilution for virus titration. Therefore, the maximum theoretical residual roseofungin concentration entering the first dilution step of the titration was 1.6 µg/mL, followed by 0.16, 0.016, 0.0016, and 0.00016 µg/mL in subsequent 10-fold dilutions.

Importantly, the maximum residual concentration of roseofungin after the first 10-fold dilution (1.6 µg/mL) was below the experimentally determined EC50 range of approximately 2.0–2.5 µg/mL. Subsequent titration dilutions resulted in progressively lower roseofungin concentrations.

Residual viral infectivity was quantified by endpoint titration and expressed as TCID_50_/mL or EID_50_/mL. Virucidal activity was reported as the reduction in infectious titer (Δlog_10_) relative to untreated virus controls [27].

### 2.19. Molecular Docking

Molecular docking studies were conducted to evaluate the potential interactions of roseofungin with selected influenza A virus proteins. Ligand preparation and structural visualization were performed using PyMOL version 3.1.4 (https://pymol.org; accessed on 8 January 2026). Docking calculations were carried out with AutoDock Vina version 1.2 [28,29], while receptor and ligand files were prepared using AutoDock Tools version 1.5.6 [30].

Three-dimensional structures of viral proteins were obtained from the Protein Data Bank (PDB) [31]. The analysis included hemagglutinin (HA; PDB IDs: 1RU7, 8G2K, 1JSD, 1TI8, and 1JSM), a glycoprotein responsible for viral attachment to host cells and subsequent membrane fusion, and neuraminidase (NA; PDB ID: 2HT5), which plays a key role in the release of newly formed virions from infected cells. To account for structural flexibility and conformational diversity, several crystallographic models of HA and NA were incorporated into the docking analysis.

The chemical structures of roseofungin (CID 6444203), rimantadine (CID 5071), and ribavirin (CID 37542) were downloaded from PubChem in SDF format. The ligands were then converted into PDB files for structural inspection and subsequently prepared in PDBQT format for docking simulations using AutoDock Vina.

### 2.20. Protonation-State Analysis

The protonation behavior of roseofungin (PubChem CID 6444203) was evaluated based on its molecular structure and predicted pKa. Roseofungin contains multiple hydroxyl groups but no basic nitrogen atoms or free carboxylic acid groups. The reported predicted pKa of approximately 14.0 indicates that deprotonation is highly unfavorable at physiological pH. At pH 7.4, the Henderson–Hasselbalch relationship predicts a deprotonated-to-neutral species ratio of approximately 2.5 × 10^−7^, corresponding to an estimated neutral fraction of >99.9999%. Therefore, the neutral microspecies was selected for molecular docking and subsequent molecular dynamics simulations.

### 2.21. In Silico Prediction of Hydrolytic Susceptibility

The hydrolytic susceptibility of the macrolactone ester moiety of roseofungin was evaluated using the U.S. Environmental Protection Agency (EPA) Chemical Transformation Simulator (CTS). The Generate Transformation Products workflow was applied using the hydrolysis reaction library with a generation limit of 1. The predicted transformation products were evaluated with respect to the intramolecular macrolactone ester bond connecting the termini of the 36-membered ring.

### 2.22. Molecular Dynamics Simulation

For molecular dynamics (MD) studies, roseofungin was chosen as the lead compound due to its favorable docking performance and favorable safety characteristics, making it a promising candidate for further investigation.

Molecular dynamics simulations of the complex based on the PDB structure 1JSM were performed using GROMACS 2024.4 with the CHARMM36m force field. System preparation and ligand parameterization were performed using the Swiss-Param program via the CHARMM-GUI interface. The monomeric protein was placed in a cubic box containing TIP3P water and 0.15 M NaCl, with a minimum distance of 1.0 nm between the protein and the box boundaries. Following energy minimization, the system was equilibrated under NVT and NPT conditions for 100 ps each at 310 K and 1 bar using the V-rescale thermostat and Parrinello–Rahman barostat.

Three independent 50 ns production MD simulations (Run 1, Run 2, and Run 3) were performed for the roseofungin–1JSM complex, resulting in a cumulative production simulation time of 150 ns. Each independent production run was performed with a 2 fs time step under periodic boundary conditions (PBC). Hydrogen-containing bonds were constrained using the LINCS algorithm. Long-range electrostatic interactions were treated using the particle mesh Ewald (PME) method with a cutoff radius of 1.2 nm. Trajectories from each independent run were processed separately using the gmx trjconv utility to remove periodicity artifacts and center the system.

The three independent trajectories were analyzed separately to assess the reproducibility and stability of the complex. Protein backbone stability was evaluated by root-mean-square deviation (RMSD), residue-level flexibility by root-mean-square fluctuation (RMSF), and ligand positional stability within the active site by RMSD of the ligand heavy atoms. Intermolecular hydrogen bonds between the ligand and the protein were analysed for each trajectory using a donor–acceptor distance criterion of <0.35 nm. All trajectory analyses were performed using GROMACS, and the resulting data from the three independent simulation runs were subsequently visualized and compared using GraphPad Prism version 10.04.0.

### 2.23. Statistical Analysis

All experiments were performed in at least three independent biological replicates unless otherwise specified.

Data are presented as mean ± standard deviation (SD). Concentration–response curves and corresponding CC_50_, EC_50_, and IC_50_ values were calculated by nonlinear regression analysis using GraphPad Prism software.

Differences among experimental groups were evaluated using one-way analysis of variance (ANOVA), followed by Tukey’s multiple-comparison test [32].

Assumptions of normality and homogeneity of variance were assessed before application of parametric statistical tests. A two-sided *p* value of less than 0.05 was considered statistically significant.

## 3. Results

### 3.1. Cytotoxicity and Safety Profile

The cytotoxicity of roseofungin was evaluated in three mammalian cell lines to establish a concentration range suitable for antiviral testing. Dose–response analyses demonstrated measurable but moderate cytotoxicity, with CC_50_ values of 50.0 ± 0.16 μg/mL in MDCK cells, 60.0 ± 0.21 μg/mL in NCTC 1469 cells, and 65.0 ± 0.23 μg/mL in PO cells (Table 1, Figure S1).

Under identical experimental conditions, no measurable cytotoxicity was observed for ribavirin at the highest concentration tested (>100 μg/mL).

Preliminary safety assessment in embryonated chicken eggs and mice indicated low acute mortality under the experimental conditions employed. The median lethal dose (LD_50_) exceeded 20 mg/kg in embryonated eggs and 25 mg/kg in mice.

Taken together, these findings established a concentration range suitable for subsequent antiviral evaluation and indicated a measurable therapeutic window for roseofungin in the experimental systems used.

### 3.2. Molecular Docking

Protonation-state analysis indicated that roseofungin is overwhelmingly neutral at physiological pH. Based on the predicted pKa of approximately 14.0, the calculated fraction of the deprotonated species at pH 7.4 is negligible (<0.001%), whereas the neutral form accounts for >99.9999% of the population. Accordingly, the neutral microspecies was used for docking and molecular dynamics simulations.

To explore potential molecular targets, docking simulations were performed using representative influenza A structural proteins. The results indicated that roseofungin exhibited higher predicted binding affinities for the studied proteins than the reference antivirals rimantadine and ribavirin. Although these findings suggest favorable binding interactions, they do not demonstrate binding selectivity or confirm biological activity.

Roseofungin showed binding energies ranging from −7.4 to −8.9 kcal/mol (Table 2), with particularly strong predicted interactions observed for hemagglutinin subtypes H1, H5, and H7, as well as neuraminidase N8.

LigPlot+ analysis identified multiple hydrogen-bond and van der Waals interactions (Table 3), including contacts with amino acid residues located within conserved regions of hemagglutinin.

These residues are known to participate in the conformational changes required for viral membrane fusion.

Overall, the molecular docking analysis predicts favorable interactions of roseofungin with the influenza A virus proteins examined. Because docking scores scale with molecular size, ligand efficiencies are reported in Table 2; on this size-normalized basis, roseofungin does not exceed the negative-control compounds, and no comparative binding claim is made. To further validate the docking-predicted binding mode and assess the stability of the protein–ligand complex under dynamic conditions, molecular dynamics simulations were performed using the hemagglutinin–roseofungin complex as a representative model.

### 3.3. Molecular Dynamics Simulation and System Stability Analysis

**Figure 2:**
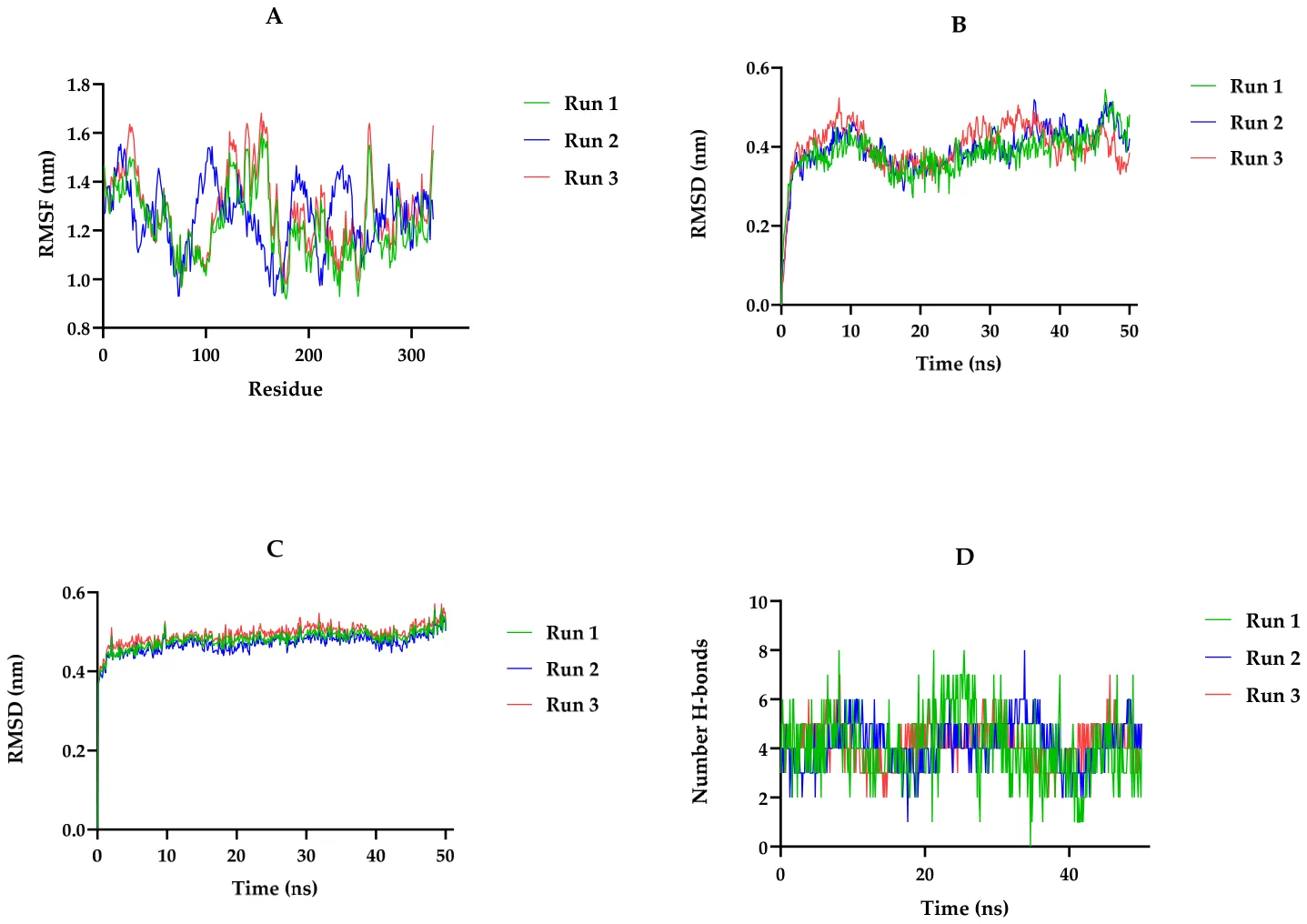
Molecular dynamics simulation analysis of the hemagglutinin–roseofungin complex across three independent 50 ns simulation runs. (**A**) Root-mean-square fluctuation (RMSF) of hemagglutinin residues. (**B**) Root-mean-square deviation (RMSD) of roseofungin heavy atoms within the predicted binding region. (**C**) Root-mean-square deviation (RMSD) of hemagglutinin backbone atoms. (**D**) Time evolution of the number of intermolecular hydrogen bonds between roseofungin and hemagglutinin. Each panel shows the results from Run 1, Run 2, and Run 3.

To evaluate the stability and reproducibility of the hemagglutinin–roseofungin complex under dynamic conditions, three independent 50 ns molecular dynamics simulations (Run 1, Run 2, and Run 3) were performed, corresponding to a cumulative production simulation time of 150 ns. All simulations were conducted under identical conditions at 310 K and 1 atm in an explicit solvent environment. The resulting trajectories were analyzed using RMSF, protein and ligand RMSD, intermolecular hydrogen-bond analysis, representative structural analysis, and residue–ligand contact occupancy analysis (Figure 2).

RMSF analysis

Residue-level flexibility of hemagglutinin was evaluated using RMSF analysis for each independent trajectory (Figure 2A). The three RMSF profiles showed a generally comparable distribution of residue fluctuations across the protein sequence. Most residues exhibited moderate fluctuations, whereas several regions displayed higher local mobility. Differences in the magnitude and position of individual fluctuation peaks were observed among the three independent runs, indicating variation in local residue dynamics between trajectories.

RMSD analysis

The structural stability of hemagglutinin was assessed by calculating the RMSD of the protein backbone atoms (Figure 2C). All three trajectories showed an initial structural adjustment followed by fluctuations around relatively stable values during the production simulations. Although the magnitude and temporal profile of the RMSD values varied between Run 1, Run 2, and Run 3, none of the trajectories exhibited a sustained increase in backbone RMSD indicative of a major structural deviation.

The RMSD of roseofungin heavy atoms within the predicted binding region was also monitored throughout the simulations (Figure 2B). The ligand RMSD profiles displayed transient fluctuations and differences between the independent runs. However, no sustained increase in ligand RMSD was observed during any of the three trajectories.

Intermolecular hydrogen-bond analysis

The number of intermolecular hydrogen bonds between roseofungin and hemagglutinin was monitored throughout each simulation (Figure 2D). Hydrogen-bond formation varied over time in all three trajectories, with differences in the number and duration of individual interactions between runs. Intermolecular hydrogen bonds were detected throughout the simulations, although their number and persistence varied between the independent trajectories.

Representative structures

**Figure 3:**
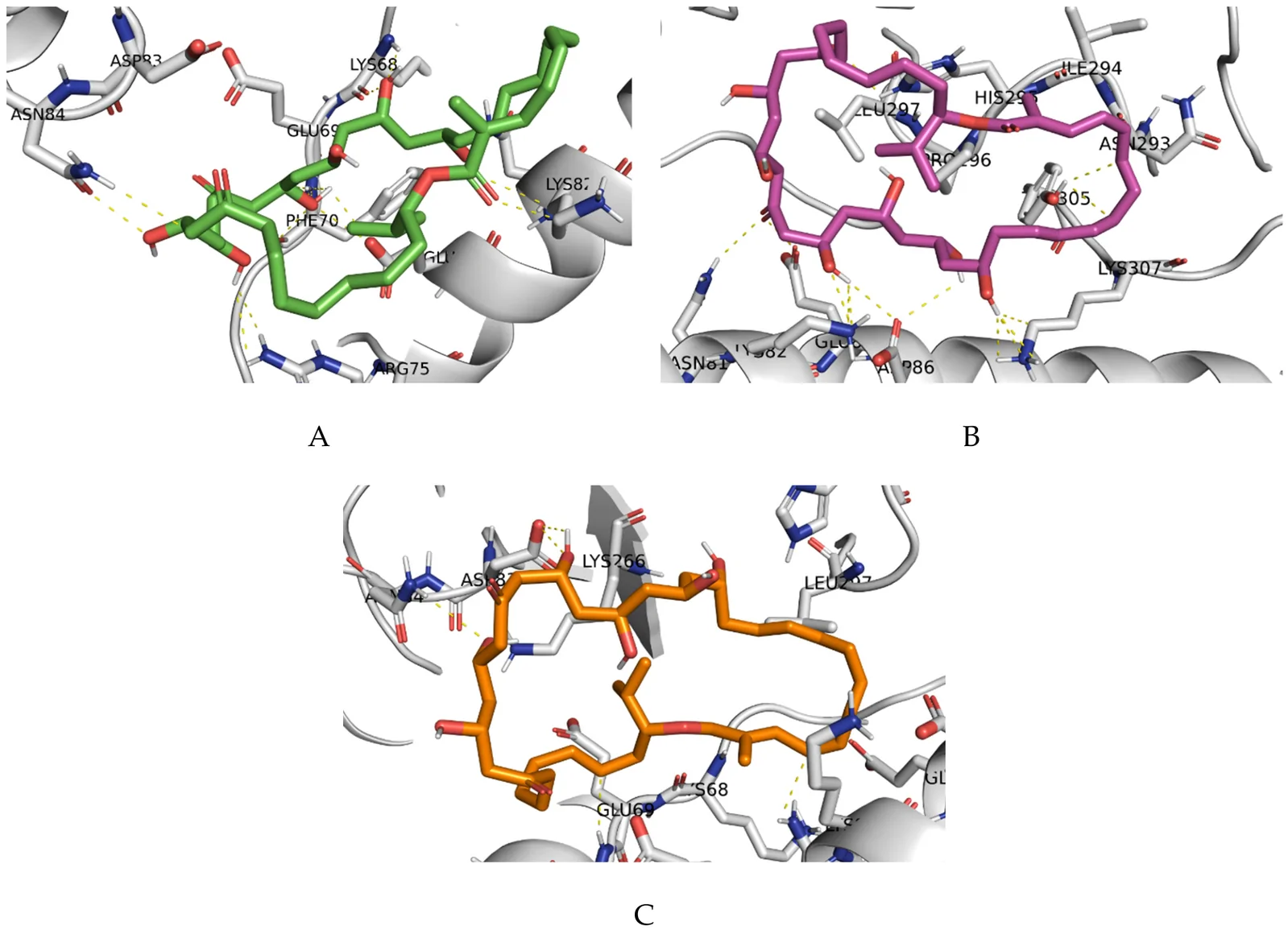
Representative roseofungin–hemagglutinin interaction patterns obtained from three independent 50 ns molecular dynamics simulations (PDB ID: 1JSM). Panels (**A**–**C**) show representative binding configurations from Run 1, Run 2, and Run 3, respectively. Roseofungin is shown in green (**A**), magenta (**B**), and orange (**C**), while hemagglutinin and surrounding amino acid residues are shown in gray. Yellow dashed lines indicate intermolecular contacts between roseofungin and residues within the predicted binding region.

Representative structures obtained from the three independent simulations are shown in Figure 3.

The overall localization of roseofungin within the predicted hemagglutinin binding region was maintained across the three representative structures, whereas differences in the orientation of the ligand and the surrounding interacting residues were observed between the runs. These structural differences were consistent with the variation in residue–ligand contacts observed across the independent trajectories.

Residue–ligand contact occupancy

**Figure 4:**
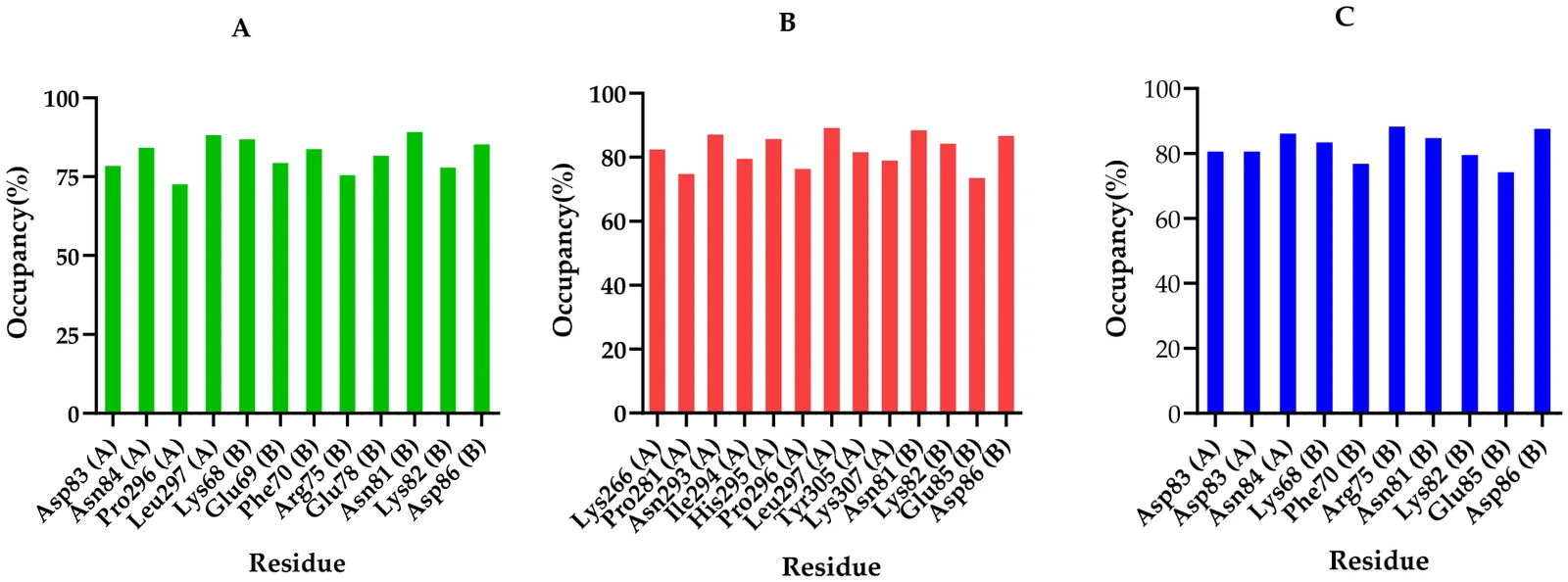
Residue–roseofungin contact occupancy profiles obtained from three independent 50 ns molecular dynamics simulations of the hemagglutinin–roseofungin complex (PDB ID: 1JSM). (**A**) Run 1; (**B**) Run 2; (**C**) Run 3. Occupancy is expressed as the percentage of analyzed trajectory frames in which each hemagglutinin residue maintained contact with roseofungin. Differences in residue composition and occupancy among the three runs reflect variability in the contact patterns sampled during the independent simulations.

Residue–ligand contact occupancy was analyzed for each of the three independent 50 ns trajectories to characterize the frequency of contacts between hemagglutinin residues and roseofungin (Figure 4).

Occupancy was expressed as the percentage of analyzed trajectory frames in which a given residue was in contact with the ligand.

In Run 1, the highest contact occupancies were observed for Asn81(B) (89.13%), Leu297(A) (88.24%), Lys68(B) (86.91%), and Asp86(B) (85.27%). In Run 2, the highest occupancies were observed for Leu297(A) (89.21%), Asn81(B) (88.46%), Asp86(B) (86.73%), His295(A) (85.72%), and Lys82(B) (84.19%). In Run 3, the highest values were recorded for Arg75(B) (88.31%), Asp86(B) (87.65%), Asn84(A) (86.14%), and Asn81(B) (84.76%).

Asn81(B), Lys82(B), and Asp86(B) were detected in all three independent trajectories. The occupancy of Asn81(B) ranged from 84.76% to 89.13%, while Lys82(B) and Asp86(B) showed occupancy ranges of 77.84–84.19% and 85.27–87.65%, respectively. Other contacts showed greater variability between the trajectories. Runs 1 and 3 shared several interactions involving Asp83(A), Asn84(A), Lys68(B), Phe70(B), and Arg75(B), whereas Run 2 showed additional contacts involving residues from chain A, particularly within the 266–307 region.

Overall, the three independent trajectories showed comparable global protein behavior, while differences in local residue fluctuations, ligand RMSD, hydrogen-bond formation, and residue–ligand contact patterns were observed between runs. The occupancy analysis identified a subset of residues that were repeatedly involved in interactions with roseofungin across the independent simulations.

### 3.4. In Silico Prediction of Hydrolytic Susceptibility

The EPA CTS analysis predicted hydrolytic opening of the 36-membered macrolactone ring of roseofungin. The predicted transformation converted the parent compound (C_39_H_62_O_10_; molecular mass, 690.915 Da) into the corresponding ring-opened hydrolysis product (C_39_H_64_O_11_; molecular mass, 708.930 Da). The predicted transformation was classified as “LIKELY” by the CTS model. These findings indicate that the macrolactone ester moiety represents a potential site of hydrolytic liability in roseofungin (Table S1).

The CTS output provides a qualitative prediction of hydrolytic transformation and does not provide a quantitative hydrolysis rate constant or half-life under defined conditions such as pH 7.4 and 37 °C. Therefore, the present in silico analysis identifies potential hydrolytic susceptibility but does not establish the time-dependent chemical stability of roseofungin under the conditions used in the antiviral assays.

### 3.5. Inhibition of Viral Adsorption

The effect of roseofungin on viral adsorption was investigated using MDCK cell cultures infected with Influenza A viruses. Viral inocula were pre-incubated with serial concentrations of roseofungin prior to exposure to cell monolayers.

**Figure 5:**
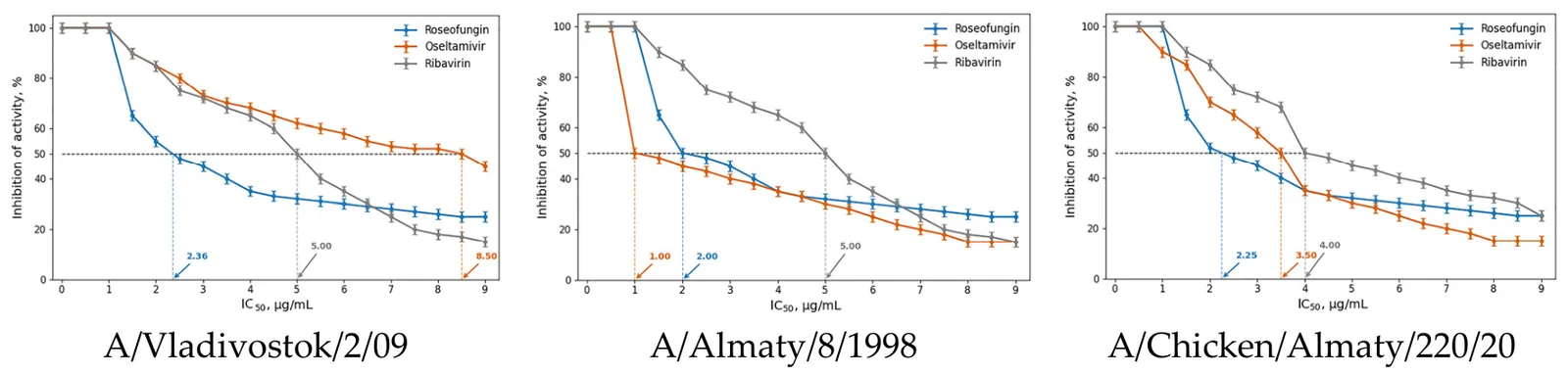
Effect of roseofungin on viral adsorption in MDCK cells.

Quantification of cell-associated viral RNA by RT-qPCR demonstrated a concentration-dependent reduction in viral adsorption (Figure 5). Ribavirin or Oseltamivir was used as a reference compound for comparative purposes.

MDCK cell monolayers were infected with Influenza A virus pre-incubated with increasing concentrations of roseofungin. Viral adsorption was quantified by RT-qPCR targeting the influenza A matrix (M) gene. Data represent mean ± SD from three independent experiments. IC_50_ values were calculated by nonlinear regression analysis.

The inhibitory effect was observed across all tested influenza strains and showed a similar concentration–response profile.

The concentration required to reduce viral adsorption by 50% (IC_50_) ranged from approximately 2.0 to 2.5 μg/mL. Although adsorption was substantially reduced at higher concentrations, residual viral RNA remained detectable in all experimental groups.

These findings indicate that roseofungin interferes with early virus–cell interactions under the conditions of the assay.

### 3.6. Neuraminidase Inhibition

The influence of roseofungin on influenza virus neuraminidase activity was examined using a fluorometric MUNANA-based assay.

**Figure 6:**
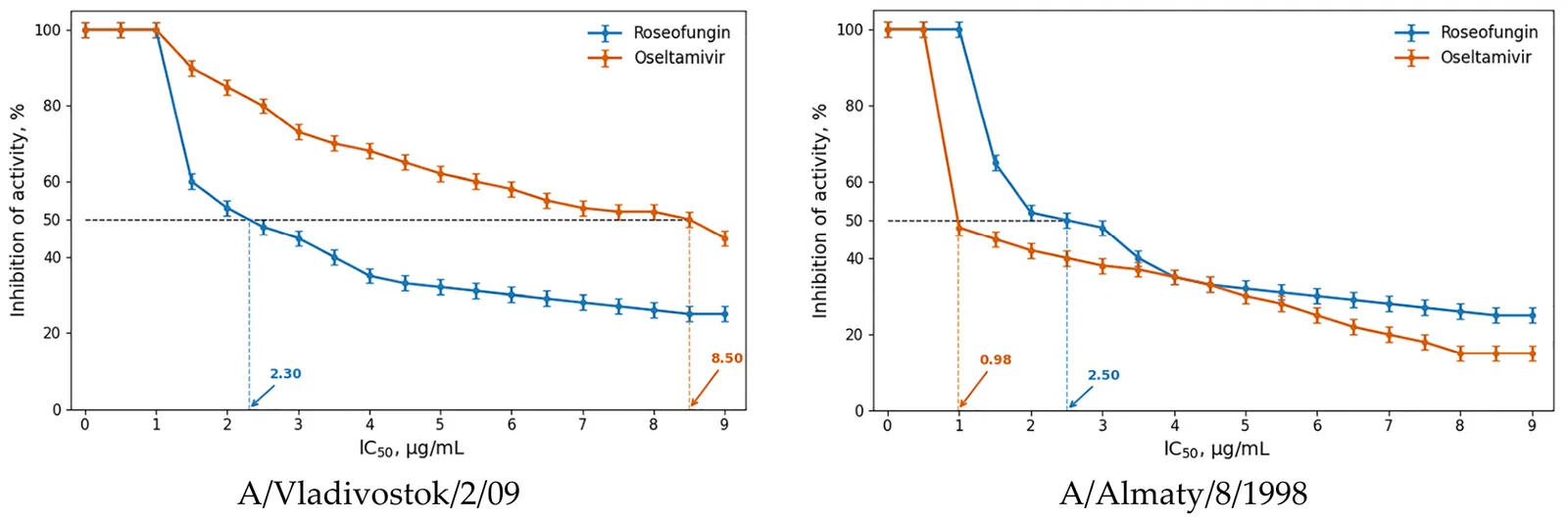
Effect of roseofungin on influenza virus neuraminidase activity.

A concentration-dependent reduction in neuraminidase activity was observed following treatment with roseofungin (Figure 6).

Neuraminidase activity was measured using a MUNANA-based fluorometric assay. Influenza A virus was incubated with serial concentrations of roseofungin prior to substrate addition. Results are expressed as percentage inhibition relative to untreated control. Data represent mean ± SD from three independent experiments. IC_50_ values were determined by nonlinear regression analysis.

The estimated IC_50_ values were within the range of 2.0–2.5 μg/mL.

Inhibition remained incomplete at the highest concentrations tested, indicating partial suppression of neuraminidase activity rather than complete enzymatic blockade.

These results demonstrate that roseofungin reduces measurable neuraminidase activity in vitro under the experimental conditions employed.

### 3.7. Antiviral Activity

The antiviral activity of roseofungin was evaluated against a panel of Influenza A virus strains representing human, swine, and avian lineages.

Roseofungin inhibited replication of all tested virus strains, with EC_50_ values ranging from 2.0 to 2.5 μg/mL (Table 4, Figure S2).

Selectivity indices varied between 20 and 25, reflecting the relationship between antiviral activity and cytotoxicity.

Comparable antiviral activity was observed across genetically diverse influenza subtypes, including H1N1, H3N2, H5N3, H7N1, and H9N2 viruses. Notably, activity was retained against the oseltamivir-resistant strain A/Vladivostok/2/09 (H1N1), for which an EC_50_ value of 2.5 ± 0.04 μg/mL was observed.

Reference antivirals exhibited the expected strain-dependent activity profiles. Oseltamivir showed reduced activity against the oseltamivir-resistant isolate, whereas roseofungin maintained comparable antiviral efficacy across the tested virus panel.

Overall, these findings demonstrate broad in vitro antiviral activity of roseofungin against multiple Influenza A virus subtypes.

### 3.8. Reduction in Viral Infectivity

The direct effect of roseofungin on viral infectivity was evaluated using a virucidal assay. Viral suspensions were exposed to 32 μg/mL roseofungin for 30 min prior to infectivity titration.

Treatment resulted in reproducible reductions in infectious viral titers across all tested Influenza A virus strains (Table 5).

Reductions ranged from 1.5 to 2.5 log_10_ TCID_50_/mL or EID_50_/mL relative to untreated controls.

The largest reductions were observed for A/Vladivostok/2/09 (H1N1) and A/Chicken/FPV/Rostock/1934 (H7N1), both of which exhibited decreases of 2.5 log_10_ units. The smallest reduction was observed for A/Chicken/Almaty/220/20 (H9N2), with a decrease of 1.5 log_10_ units.

Despite differences in magnitude, all tested strains demonstrated statistically significant reductions in infectivity following treatment.

These data indicate that exposure to roseofungin decreases the recoverable infectivity of Influenza A virus particles under the assay conditions.

## 4. Discussion

The continued emergence of antiviral resistance and the limited number of available therapeutic options highlight the need for new antiviral agents with alternative mechanisms of action [6,9]. In the present study, roseofungin demonstrated reproducible antiviral activity against a diverse panel of influenza A virus strains, including an oseltamivir-resistant isolate. Antiviral effects were observed across multiple experimental systems, indicating that the activity of roseofungin was not restricted to a particular virus strain or experimental model.

The antiviral potency observed in the present study was characterized by EC_50_ values ranging from 2.0 to 2.5 μg/mL and selectivity indices of approximately 20–25. Although these parameters do not predict clinical efficacy, they demonstrate measurable antiviral activity at concentrations below those associated with substantial cytotoxicity under the experimental conditions used. Comparable activity against human, swine, and avian influenza A virus strains further suggests that the observed antiviral effect is not strongly restricted by the genetic diversity represented by the tested strains.

An important finding was the retention of antiviral activity against the oseltamivir-resistant strain A/Vladivostok/2/09 (H1N1). Resistance to neuraminidase inhibitors remains an important concern in influenza treatment because mutations affecting drug susceptibility may emerge during antiviral therapy or circulate naturally in viral populations [33]. The comparable activity of roseofungin against susceptible and oseltamivir-resistant viruses suggests that its antiviral effect is unlikely to depend exclusively on the molecular determinants responsible for oseltamivir susceptibility. However, this observation does not identify the molecular target of roseofungin and does not exclude mechanisms involving either viral or cellular components.

Functional assays showed that roseofungin reduced viral adsorption and partially decreased neuraminidase-associated activity. These findings indicate that the compound can affect processes relevant to influenza virus attachment and/or release. Nevertheless, reduced viral adsorption does not necessarily indicate direct binding to hemagglutinin (HA), as it may result from direct effects on virions, alterations in virus–cell interactions, or indirect effects on membrane-associated processes [34,35]. Similarly, the reduction in neuraminidase activity does not by itself establish direct enzymatic inhibition, because changes in virion structure or organization may also affect the accessibility or activity of the enzyme in the experimental system [36]. Thus, the functional assays provide evidence that roseofungin affects viral adsorption and neuraminidase-associated activity, while the molecular basis of these effects remains to be established.

The molecular mechanism underlying the antiviral activity of roseofungin was therefore further explored using molecular docking. The computational analysis was designed as a comparative and hypothesis-generating investigation of potential interactions between roseofungin and influenza A surface proteins, rather than as a target-validation assay. Several experimentally determined HA structures representing different influenza A subtypes were investigated to determine whether roseofungin could establish predicted interactions with structurally distinct HA proteins. The N8 neuraminidase structure (PDB 2HT5) was additionally included intentionally as a second class of influenza surface glycoprotein. Thus, 2HT5 was not considered an HA structure or an HA positive control, but was used to explore whether the predicted interaction capacity of roseofungin could extend beyond HA to another major surface protein of influenza virus.

The predicted interaction patterns differed among the investigated HA structures. The LigPlot analyses demonstrated that roseofungin could form hydrogen-bonding and other non-covalent contacts with multiple residues located in different regions of HA. These interactions should therefore not be interpreted as evidence that roseofungin specifically occupies the canonical sialic-acid receptor-binding site of HA. Rather, the docking results indicate that several surface regions of influenza HA may be capable of accommodating the roseofungin molecule under the computational conditions used. This interpretation is consistent with the exploratory nature of the analysis and avoids assigning a specific molecular target in the absence of direct biochemical or biophysical validation.

Ribavirin and rimantadine were included in the docking analysis as reference antiviral small molecules. They were not intended to serve as experimentally validated positive controls for HA binding. Their established antiviral mechanisms do not imply direct interaction with the HA structures investigated in the present study. Consequently, docking scores obtained for these compounds should not be interpreted as measures of their clinical antiviral efficacy or as tests of their established mechanisms of action. The purpose of including these compounds was to provide a comparative small-molecule reference within the same computational framework. Accordingly, the computational results should be interpreted independently of the known clinical activity of these reference drugs.

The distinction between comparative docking and experimental target validation is important. Positive controls such as receptor-binding glycans, fetuin, antibodies, antisera, or reference viruses are appropriate for specific biochemical or virological HA receptor-binding or inhibition assays. These systems, however, are not directly equivalent to small-molecule ligands in a molecular docking experiment because they address different molecular interactions and experimental endpoints. A small molecule with an experimentally determined binding mode to the same HA region would represent a more direct structural reference for docking validation. The absence of such a target-specific structural control is therefore recognized as a limitation of the present computational analysis.

To further examine the reproducibility of the predicted HA–roseofungin interaction under dynamic conditions, three independent 50 ns molecular dynamics simulations were performed. The trajectories showed comparable overall protein RMSD and RMSF behavior, although differences in local fluctuations were observed between independent runs. Similarly, the ligand RMSD profiles exhibited run-specific fluctuations without a sustained increase indicative of progressive displacement from the predicted interaction region. These results suggest that the overall conformational behavior of the complex was reproducible across the independent simulations, while retaining the local variability expected for molecular dynamics trajectories.

The intermolecular hydrogen-bond analysis further demonstrated that interactions between roseofungin and HA were maintained dynamically during the simulations, although the number and persistence of individual hydrogen bonds varied between trajectories. This behavior is consistent with a dynamic protein–ligand interaction network rather than a single rigid binding configuration. Representative structures obtained from the three simulations showed preservation of the overall localization of roseofungin within the predicted interaction region, accompanied by variations in ligand orientation and local residue contacts.

Residue–ligand contact occupancy analysis identified several residues that repeatedly interacted with roseofungin across the independent trajectories. In particular, Asn81, Lys82, and Asp86 were detected in all three simulations and showed relatively high occupancy, whereas other interactions varied between trajectories. The presence of recurrent contacts, together with trajectory-specific interactions, suggests that the predicted interaction region may retain a degree of structural persistence while allowing local adaptability of the ligand and surrounding residues. These residues therefore represent candidates for future experimental investigation. However, their functional contribution to antiviral activity remains unknown and should not be inferred solely from the computational results.

Importantly, the molecular dynamics simulations provide additional computational support for the reproducibility of the predicted HA–roseofungin interaction, but they do not establish HA as the primary molecular target of roseofungin. HA was selected for molecular dynamics analysis because it provided a reproducible predicted interaction suitable for further computational characterization, rather than because HA had been established experimentally as the molecular target. The experimental findings involving viral adsorption and neuraminidase-associated activity, together with the docking results obtained for multiple influenza surface proteins, indicate that several molecular mechanisms remain possible.

Roseofungin belongs to the polyene macrolide family, whose members are characterized by interactions with membrane sterols and the ability to alter membrane organization and permeability. These physicochemical properties provide a plausible additional explanation for activity against enveloped viruses. Because the influenza virus envelope is derived from host cell membranes and is involved in attachment, fusion, assembly, and release, changes in envelope integrity or membrane-associated processes could potentially affect viral infectivity. The reduction in viral infectivity observed in the virucidal assay is compatible with such a possibility. However, membrane structure, lipid composition, sterol interactions, and virion morphology were not directly investigated in the present study. Therefore, membrane-mediated mechanisms remain hypothetical and require experimental validation.

An additional consideration is the potential relationship between antiviral activity and cytotoxicity. Polyene compounds are inherently membrane-active molecules, and their biological activity may be accompanied by effects on the host cells. In the present study, roseofungin exhibited moderate cytotoxicity in cultured cells, whereas acute toxicity studies demonstrated low mortality under the tested in vivo conditions. Although these findings suggest the presence of a measurable experimental therapeutic window, they should be interpreted cautiously. Acute mortality provides only limited information regarding systemic safety, and additional studies addressing organ toxicity, histopathology, pharmacokinetics, and repeated-dose exposure are required [37].

The chemical stability of roseofungin also represents an important consideration when interpreting the antiviral experiments and computational results. Although physicochemical and solvent-related characteristics of roseofungin have been investigated previously [19,20], its stability under the exact conditions used in the present antiviral assays, including the concentration of 32 μg/mL, an ethanol-containing medium, and incubation at 37 °C, was not independently determined in this study. In particular, the presence of an ester group raises the possibility of hydrolytic transformation under certain conditions. To further assess this possibility, an in silico prediction was performed using the U.S. Environmental Protection Agency (EPA) Chemical Transformation Simulator (CTS). The Generate Transformation Products workflow was applied using the Hydrolysis reaction library with a generation limit of 1. The CTS model predicted hydrolytic opening of the 36-membered macrolactone ring, converting the parent compound (C_39_H_62_O_10_; molecular mass, 690.915 Da) into the corresponding ring-opened hydrolysis product (C_39_H_64_O_11_; molecular mass, 708.930 Da). The predicted transformation was classified as “LIKELY” by the CTS model, indicating that the macrolactone ester moiety represents a potential site of hydrolytic chemical instability. However, the CTS model provides a qualitative prediction of chemical transformation and does not provide a quantitative hydrolysis rate constant or half-life at pH 7.4 and 37 °C. Consequently, the extent and rate of hydrolysis during the 30 min pre-incubation and 48 h antiviral assays cannot be inferred from the CTS prediction alone. Direct analytical monitoring of roseofungin integrity under the assay conditions would therefore be required to determine its actual chemical stability. Accordingly, the biological activity observed in the present study cannot be assumed to arise exclusively from intact roseofungin until its chemical stability under the experimental conditions has been experimentally established.

Although roseofungin has been developed and investigated as an antifungal medicinal agent, its established antifungal activity and clinical use cannot be directly extrapolated to antiviral efficacy. The concentrations effective against influenza A virus in vitro should not be interpreted as clinically achievable antiviral concentrations without appropriate pharmacokinetic and pharmacodynamic evidence. Formulation, route of administration, tissue distribution, local exposure, and duration of exposure may substantially influence antiviral efficacy. Consequently, the relationship between the experimentally determined antiviral concentrations and clinically relevant exposure remains to be established.

Taken together, the present findings provide evidence that roseofungin possesses antiviral activity against influenza A viruses and affects viral processes associated with adsorption and neuraminidase-associated activity. The computational analyses further identify several potential interactions between roseofungin and influenza surface proteins and demonstrate reproducibility of a representative HA–roseofungin interaction in independent molecular dynamics simulations. However, these computational findings should be regarded as structural hypotheses rather than definitive evidence of direct target engagement. The available data do not establish a single molecular target and remain compatible with direct interactions with viral proteins, effects on virion-associated structures, membrane-mediated mechanisms, or combinations of viral and host cell effects.

Limitations

Several limitations of this study should be acknowledged. First, the antiviral experiments were conducted in cell culture and embryonated egg systems, which do not fully reproduce the complexity of influenza virus infection in vivo. Second, although the experimental findings provide evidence for antiviral activity, the precise molecular target and mechanism of action of roseofungin remain to be established. In particular, the effects on viral adsorption and neuraminidase-associated activity should not be interpreted as evidence that neuraminidase represents the primary molecular target. Third, the molecular docking and molecular dynamics analyses provide computational evidence regarding potential protein–ligand interactions but cannot substitute for direct biochemical or biophysical target validation studies. The use of ribavirin and rimantadine as reference antiviral compounds does not constitute target-specific validation of HA binding. Fourth, the chemical stability of roseofungin under the specific conditions used in the antiviral assays was not experimentally determined. Finally, the pharmacokinetic properties, systemic exposure, and antiviral efficacy of roseofungin in relevant animal models have not been comprehensively established.

Future directions

Future studies should focus on defining the molecular and cellular mechanisms underlying the antiviral activity of roseofungin using complementary virological, biochemical, and biophysical approaches. Time-of-addition experiments will help determine which stages of the influenza virus replication cycle are most strongly affected. Target-oriented biochemical and target-engagement studies should investigate whether neuraminidase, hemagglutinin, other viral proteins, or host cell factors contribute to the observed antiviral phenotype. In particular, experimentally determined small-molecule–HA complexes or direct binding assays could be used to validate the predicted HA interactions. Mutational analysis of residues identified by the molecular dynamics contact-occupancy analysis, including Asn81, Lys82, and Asp86, may further determine whether these residues contribute functionally to the predicted interaction.

In parallel, membrane interaction and biophysical studies, together with lipidomic profiling, could determine whether alterations in membrane composition, organization, or function contribute to the antiviral effect. High-resolution imaging, including electron microscopy, may provide additional evidence regarding potential effects on viral morphology, membrane integrity, or virus–cell interactions.

Further studies should also assess the chemical stability, pharmacokinetic behavior, tissue distribution, and toxicity of roseofungin to establish an appropriate exposure–response relationship. Evaluation in relevant animal models of influenza infection will be essential to determine whether the antiviral activity observed in vitro and in ovo translates into therapeutic efficacy in vivo. Combination studies with clinically approved influenza antivirals, including oseltamivir, should determine whether roseofungin produces additive or synergistic effects and whether such combinations may be useful against drug-resistant influenza strains. A systematic head-to-head comparison of roseofungin with other polyene antibiotics under standardized experimental conditions would also be of interest.

## 5. Conclusions

In conclusion, the present findings demonstrate that roseofungin reproducibly inhibits influenza A virus infection under the experimental conditions investigated and retains antiviral activity against an oseltamivir-resistant strain. Functional assays indicate effects on viral adsorption and neuraminidase-associated activity, while molecular docking and molecular dynamics simulations provide structural hypotheses regarding potential interactions with viral proteins. Importantly, the present study does not establish neuraminidase as the primary or exclusive molecular target of roseofungin. Rather, the findings support the possibility of a multifactorial antiviral mechanism that may involve viral proteins, virion-associated structures, membrane-associated processes, and host cell factors. Further biochemical, biophysical, pharmacological, and in vivo studies will be required to define the precise mechanism of action and determine the therapeutic potential of roseofungin as an antiviral lead compound.

## Figures and Tables

**Table 1:** Comparative cytotoxicity and acute toxicity of roseofungin and ribavirin.

Ribavirin	Roseofungin	Biological Model
		Cell Lines (CC_50_)
>100 μg/mL	50.0 ± 0.16 μg/mL	MDCK (Canine Kidney)
>100 μg/mL	60.0 ± 0.21 μg/mL	NCTC 1469 (Mouse Liver)
>100 μg/mL	65.0 ± 0.23 μg/mL	PO (Sheep Kidney)
		Animal Models (LD_50_)
>30 mg/kg	>20 mg/kg	Chicken Embryos (LD_50_)
>55 mg/kg	>25 mg/kg	Mice (LD_50_)

Cytotoxicity of roseofungin was evaluated in MDCK, NCTC 1469, and PO cell lines using the MTT assay. Acute toxicity was assessed in embryonated chicken eggs and mice. CC_50_ values are expressed as mean ± SD from three independent experiments. LD_50_ values were estimated using the Reed–Muench method. Ribavirin was used as a reference compound for comparative purposes.

**Table 2:** Molecular docking binding affinities and ligand efficiencies of roseofungin and reference drugs with established antiviral activity against representative influenza A virus proteins.

Compound	Formula	Heavy Atoms	1JSD ^5^	1TI8 ^6^	2HT5 ^4^	1JSM ^3^	8G2K ^2^	1RU7 ^1^
Roseofungin	C_39_H_62_O_10_	49	−7.4/−0.151	−7.9/−0.161	−8.7/−0.178	−7.7/−0.157	−7.2/−0.147	−8.9/−0.182
Ribavirin	C_8_H_12_N_4_O_6_	18	−5.7/−0.317	−7.3/−0.406	−6.4/−0.356	−5.7/−0.317	−6.0/−0.333	−5.2/−0.289
Rimantadine	C_12_H_21_N	13	−4.5/−0.346	−6.8/−0.523	−5.8/−0.446	−5.1/−0.392	−5.5/−0.423	−5.1/−0.392

Values are binding energy (kcal/mol)/ligand efficiency (kcal/mol per heavy atom). ^1^ 1RU7, hemagglutinin H1; ^2^ 8G2K, hemagglutinin H3; ^3^ 1JSM, avian hemagglutinin H5; ^4^ 2HT5, neuraminidase N8 (apo, group 1); ^5^ 1JSD, hemagglutinin H9; ^6^ 1TI8, hemagglutinin H7. Ligand efficiency is calculated as ΔG divided by the number of non-hydrogen atoms. Ribavirin and rimantadine were used as negative controls for HA/NA binding because their established molecular targets are different viral proteins.

**Table 3:** Hydrogen-bond and van der Waals interactions between roseofungin and viral proteins.

Protein	H Bond	Van Der Waals Force	2D Visualization in LigPlot
1TI8	His283(A) Gly285(A) Ile297(A) Asn298(A) Tyr308(A) Tyr308(A) Asn296(A)	Ser284(A) Ser284(A) Man341(A) Man342(A) Ndg340(A) Glu312(A) Arg45(A) Asn47(A)	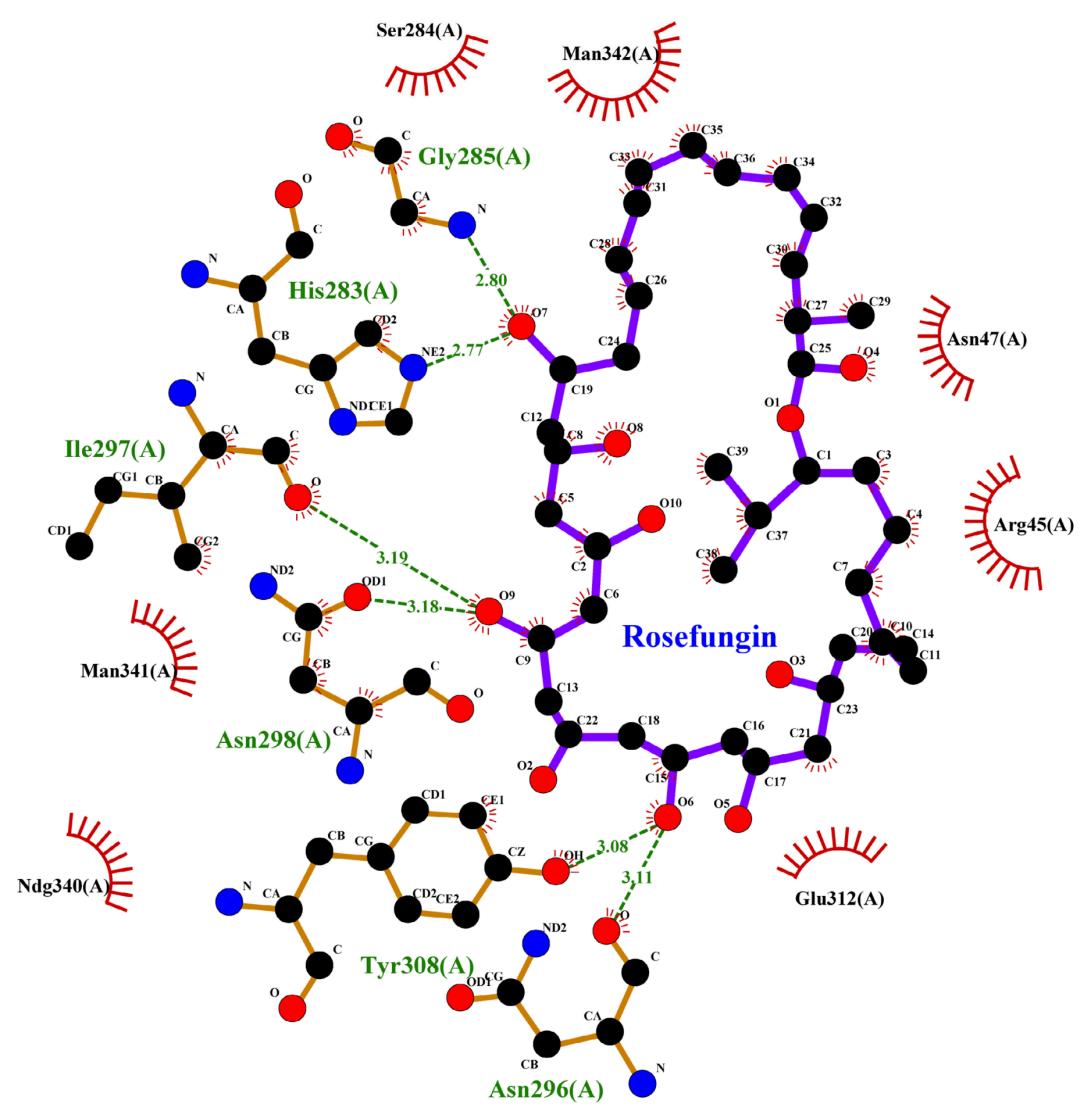
8G2K	Ser114(A), Phe63(B), His64(B), Phe63(B)	Glu81(B), Val84(B), Ile66(B), Gln65(B), Lys62(B), Gly263(A)	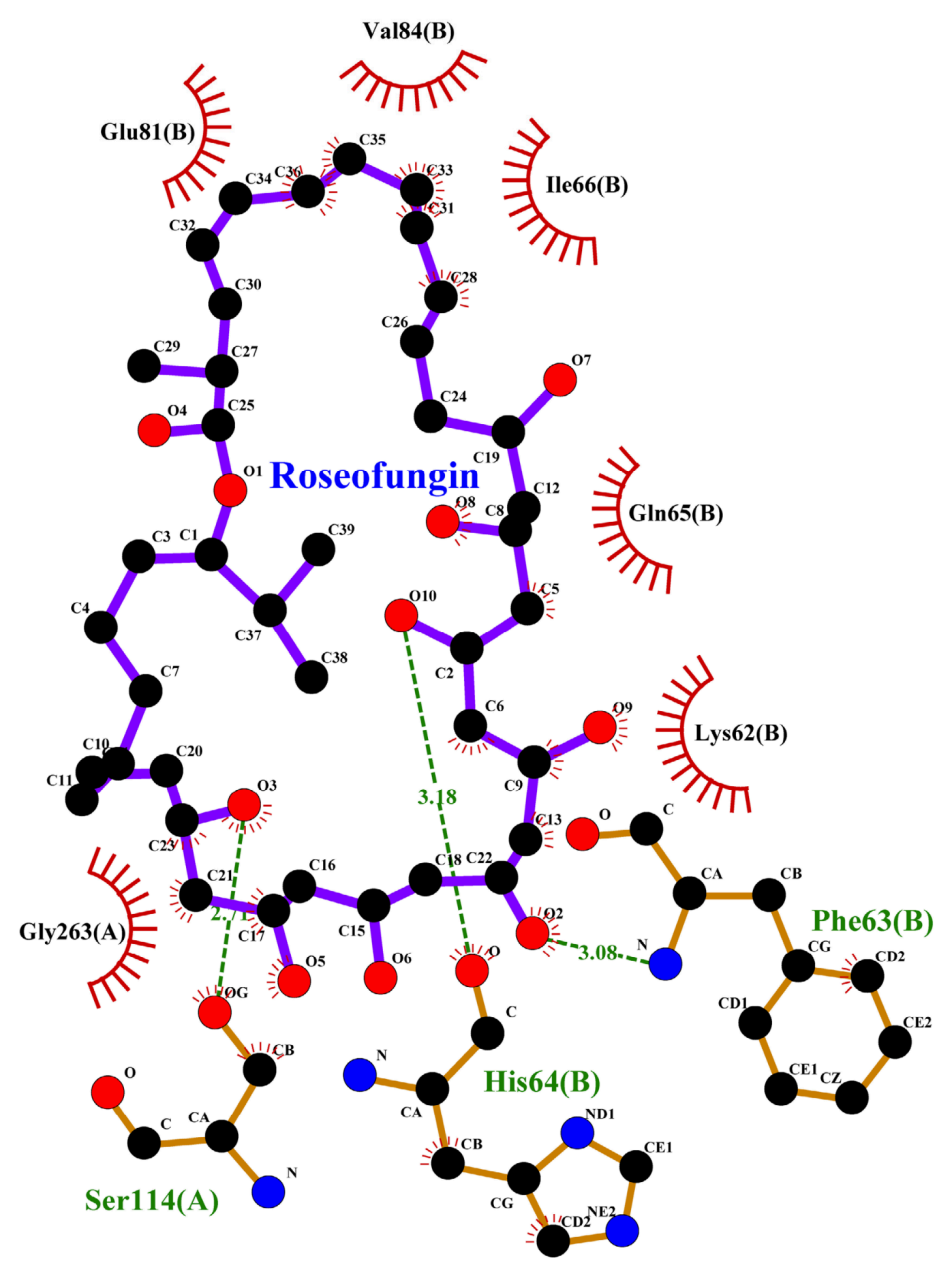
1RU7	Arg224(C), Ser207(E)	Ala62(C), Ala62(C), Phe99(C), Ile93(C), Asn60(C), Glu90(C), Asn208(E), Val223(C)	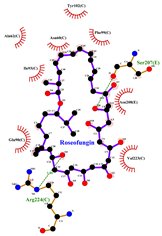
1JSD	Gly294(A), Gln62(B), His255(A)	Asn60(B), Cys296(A), Lys61(B), Asn295(A), Leu46(A), Ser254(A), Arg257(A), Cys296(A)	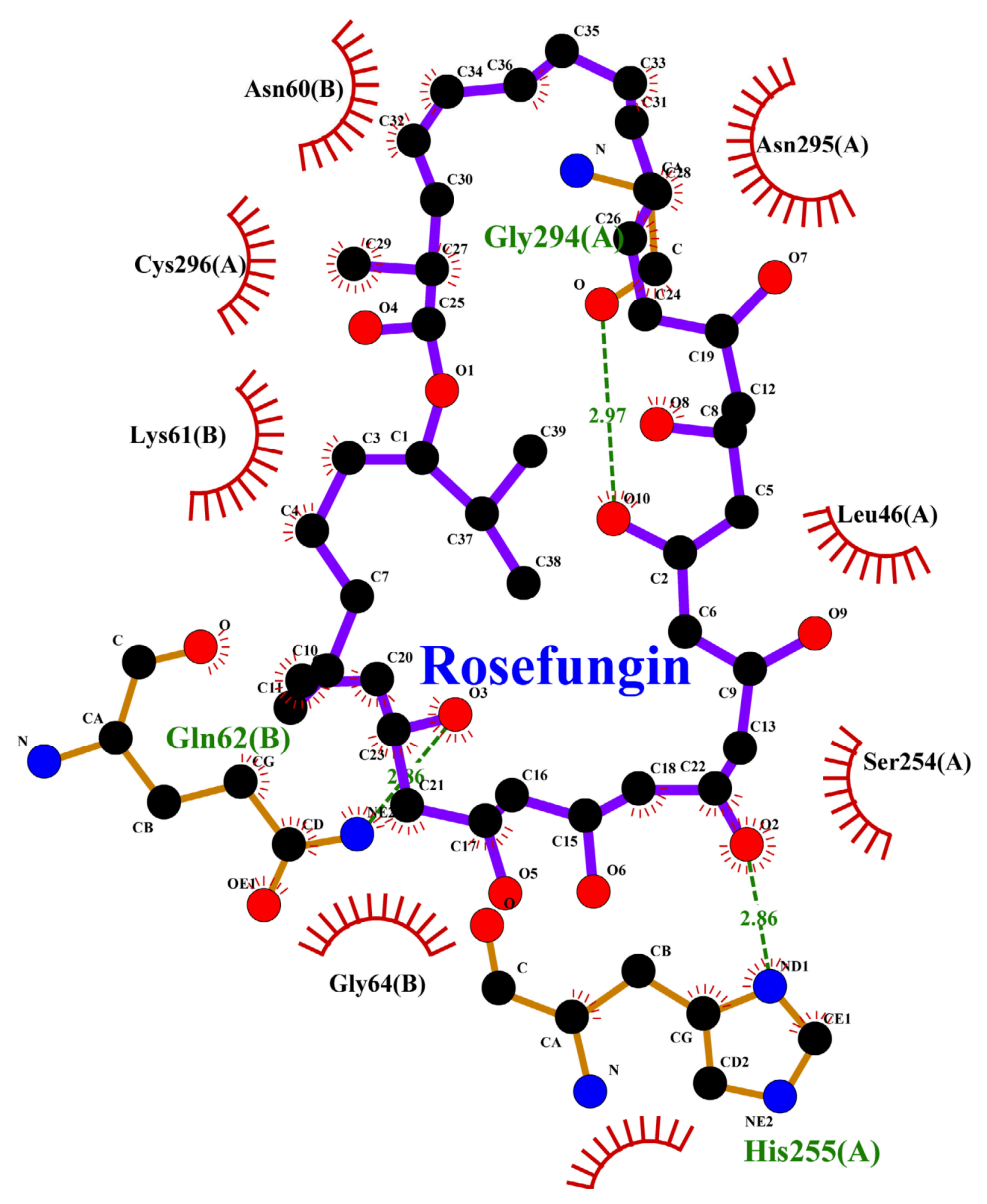
1JSM	Asn81(B), Lys68(B), Glu78(B), Phe70(B)	Asn84(A), Lys82(B), Pro296(A), Asp86(A)	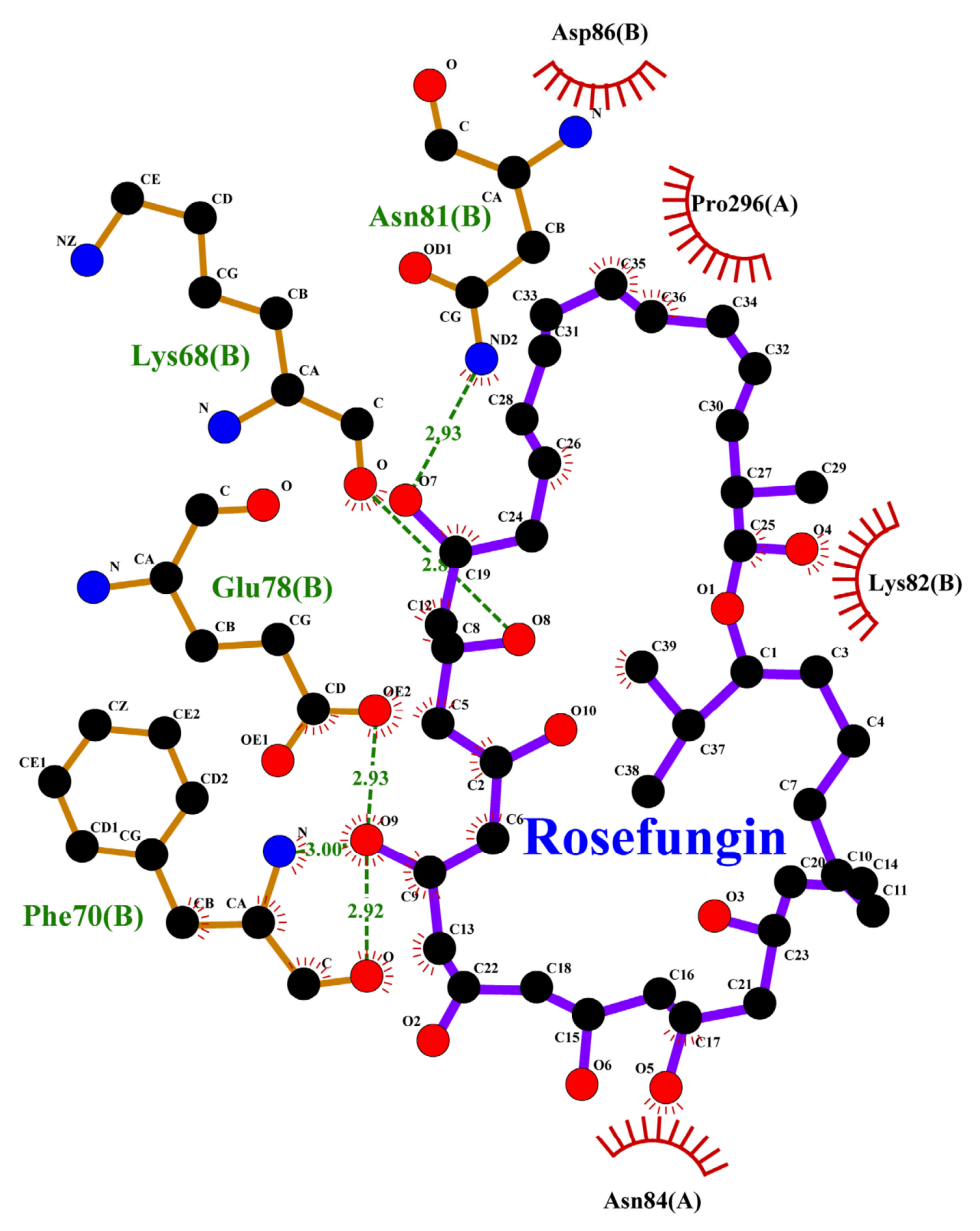
2HT5	Tyr91(B), Glu83(D), Arg51(D)	Arg51(C), Glu83(C), Arg94(A), Tyr91(A), Gln86(A), Glu83(A), Arg94(D), Tyr91(D), Tyr91(D), Asn92(B), Gln86(D), Asn92(C)	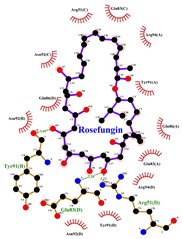

**Table 4:** Comparative inhibitory efficacy of roseofungin and clinical reference antivirals against Influenza A virus subtypes.

Virus Strain	Ribavirin EC_50_ (SI)	Rimantadine EC_50_ (SI)	Oseltamivir EC_50_ (SI)	Roseofungin EC_50_ (SI)
A/Vladivostok/2/09 (H1N1)	5.55 ± 0.03 (>18) *	4.0 ± 0.03 (>25) *	9.09 ± 0.04 (>11) *	2.5 ± 0.04 (20) *
A/Almaty/8/98 (H3N2)	4.76 ± 0.03 (>21) *	3.7 ± 0.04 (>27) *	1.33 ± 0.03 (>75) *	2.17 ± 0.08 (23) *
A/Almaty/04/19 (H1N1)	4.55 ± 0.03 (>22) *	3.33 ± 0.04 (>30) *	1.33 ± 0.03 (>75) *	2.27 ± 0.03 (22) *
A/Swine/Iowa/1/30 (H1N1)	4.17 ± 0.08 (>24) *	4.35 ± 0.02 (>23) *	2.0 ± 0.03 (>50) *	2.0 ± 0.05 (25) *
A/Tern/SA/1/61 (H5N3)	4.76 ± 0.10 (>21) *	5.0 ± 0.04 (>20) *	1.65 ± 0.03 (>60) *	2.27 ± 0.04 (22) *
A/Chicken/FPV/Rostock/34 (H7N1)	5.0 ± 0.08 (>20) *	5.56 ± 0.03 (>18) *	2.0 ± 0.03 (>50) *	2.38 ± 0.03 (21) *
A/Chicken/Almaty/220/20 (H9N2)	4.76 ± 0.11 (>21) *	4.35 ± 0.03 (>23) *	4.0 ± 0.03 (>25) *	2.27 ± 0.03 (22) *

* EC_50_ and selectivity index (SI = CC_50_/EC_50_) values were determined for roseofungin, oseltamivir, rimantadine, and ribavirin across multiple Influenza A virus strains of human, swine, and avian origin. Data are presented as mean ± SD from three independent experiments. SI values for reference compounds were calculated using CC_50_ > 100 μg/mL, as no cytotoxicity was observed at the highest tested concentration.

**Table 5:** Effect of roseofungin on influenza virus infectivity (virucidal assay).

Virus Strain	Titer Reduction (Δlog_10_)	Post-Treatment Titer (log_10_)	Baseline Titer (log_10_)
A/Vladivostok/2/09 (H1N1)	2.5	5.5 ± 0.02 *	8.0 ± 0.02
A/Almaty/8/98 (H3N2)	2	5.75 ± 0.02 *	7.75 ± 0.04
A/Almaty/04/19 (H1N1)	2	3.54 ± 0.02 *	5.54 ± 0.02
A/Swine/Iowa/1/30 (H1N1)	2	6.5 ± 0.02 *	8.50 ± 0.03
A/Tern/SA/1/61 (H5N3)	2	6.31 ± 0.03 *	8.31 ± 0.02
A/Chicken/FPV/Rostock/34 (H7N1)	2.5	6.3 ± 0.03 *	8.8 ± 0.03
A/Chicken/Almaty/220/20 (H9N2)	1.5	3.4 ± 0.03 *	4.9 ± 0.02

* Influenza A virus suspensions were incubated with 32 μg/mL roseofungin for 30 min at 37 °C prior to infectivity titration. Viral titers are expressed as log_10_ TCID_50_/mL or EID_50_/mL. Reduction in infectivity (Δlog_10_) represents the difference between treated and untreated virus samples. Data are presented as mean ± SD from three independent experiments.

## Data Availability

All the data generated or analyzed during this study are included in the published article.

## Supplementary Materials

The following supporting information can be downloaded at https://www.mdpi.com/article/10.3390/v18090941/s1: Figure S1. Dose–response curves used for the determination of IC50 values of roseofungin in MDCK (a), NCTC (b), and PO (c) cell cultures; Figure S2. Dose–response curves used for the determination of CC50 values of roseofungin and reference antiviral drugs against influenza virus model strains A/Vladivostok/2/09 (H1N1) (a), A/Almaty/8/98 (H3N2) (b), and A/Chicken/FPV/Rostock/34 (H7N1) (c). Table S1. In silico prediction of hydrolytic transformation of roseofungin using the EPA Chemical Transformation Simulator.
