# Supplementary material for: Antiviral Activity of the Polyene Macrolide Roseofungin Against Influenza a Virus: In Vitro, In Ovo, and Preliminary In Vivo Evaluation
Source: Viruses. 2026 Aug 28;18(9):941. doi: 10.3390/v18090941 (PMC13611850; doi:10.3390/v18090941)
Supplement: Supplementary file 1 [file viruses-18-00941-s001.zip › viruses-4474929-supplementary.pdf]

A

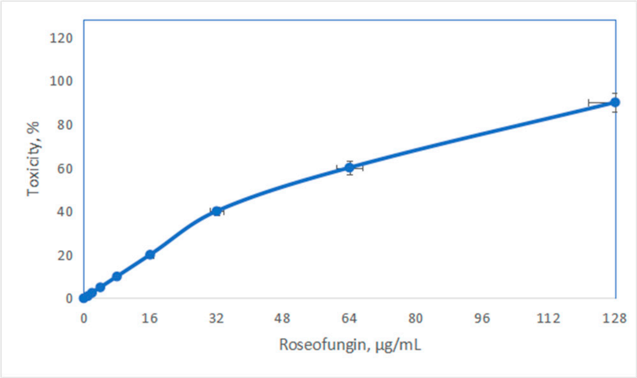

B

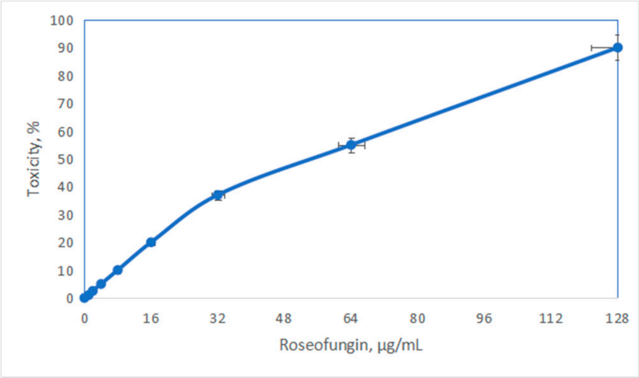

C

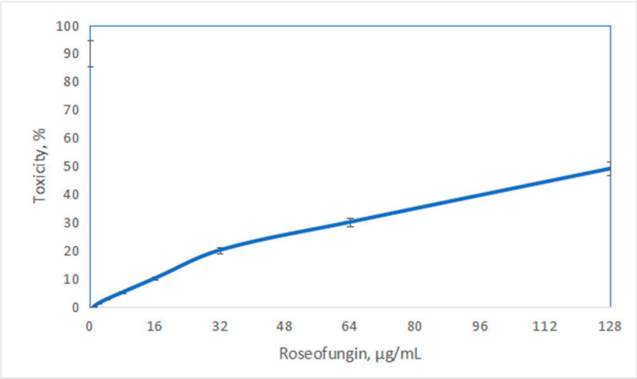

Figure S1. Dose–response curves used for the determination of IC50 values of roseofungin in MDCK (a), NCTC(b), and PO(c) cell cultures.

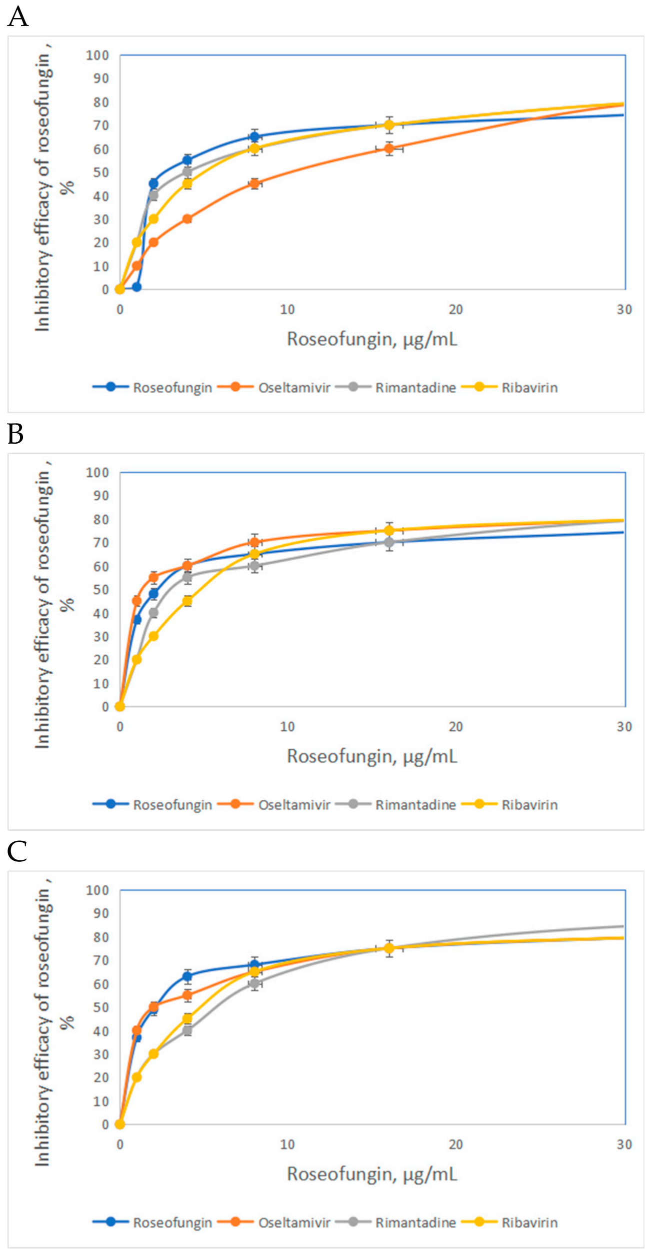

Figure S2 Dose–response curves used for the determination of CC50 values of roseofungin and reference antiviral drugs against influenza virus model strains A/Vladivostok/2/09 (H1N1)(a), A/Almaty/8/98 (H3N2)(b), and A/Chicken/FPV/Rostock/34 (H7N1)(c).

Table S1. In silico prediction of hydrolytic transformation of roseofungin using the EPA Chemical Transformation Simulator

Parameter EPA	**CTS prediction**
Compound	Roseofungin
Parent formula	$C_{39}H_{62}O_{10}$
Parent molecular mass	690.915 Da
Transformation	Lactone hydrolysis / macrolactone ring opening
Product formula	$C_{39}H_{64}O_{11}$
Product molecular mass	708.930 Da
Reaction library	Hydrolysis

Generation limit	1
CTS classification	**LIKELY**
Quantitative hydrolysis half-life	Not provided by CTS
